# Dr. Wood’s Treatment of Scarlet Fever

**Published:** 1853-04

**Authors:** 


*Dr. Wood's Treatment of Scarlet Fever.* By Prof. J. H. Bennett, Edinburgh.

The most recent system of treatment which has been brought forward is that recommended by Dr. Andrew Wood; and I notice it in deference to the great experience that gentleman has acquired from his position as physician to Heriot's Hospital and other educational establishments in the city, which have been attacked by numerous epidemics of the disease. He considers that the most efficient and safe method of treatment consists in acting powerfully on the skin, with a view of thereby assisting nature to eliminate the scarlatinal poison from the system. As ordinary diaphoretics frequently fail, he has recourse to the following method: Several common beer bottles, containing very hot water, are placed in long worsted stockings, or long narrow flannel bags, wrung out of water as hot as it can be borne. These are to be laid alongside the patient, but not in contact with the skin. One on each side, and one between the legs, will generally be sufficient; but more may be used if deemed necessary. The patient is to lie between the blankets (the head of course being outside) during the application of the bottles, and for several hours afterward. In the course of from ten minutes to a half an hour, the patient is thrown into a most profuse perspiration, when the stockings may be removed. In mild cases, the effect is easily kept up by means of draughts of cold water, and, if necessary, by the use of two dram doses of sp. mindereri, every two hours. In severe cases, where the pulse is very rapid—the beats running into each other—where the eruption is either absent or only partial, or of a dusky purplish hue—where the surface is cold —where there is sickness or tendency to diarrhœa—where the throat is aphthous or ulcerated, and the cervical glands swollen, then he follows up the use of the vapor-bath by four or five grain doses of carbonate of ammonia, repeated every three or four hours. Should this be vomited, then brandy may be given in doses proportioned to the age of the patients. Carbonate of ammonia he considers to act beneficially: 1st, by supporting the powers of life; 2d, by assisting the development of the eruption; and, 3d, by acting on the skin and kidneys. Where the vapor-bath was used early in the disease, and its use continued daily, or twice or thrice a day, according to circumstances, he has found that the chance of severe sore throat was greatly obviated. In regard to supervening dropsy, he considers that, by the use of the vapor-bath, with the other necessary precautions as to exposure, diet, etc., its recurrence is rendered much more rare. In the treatment of the dropsical cases, it was also very useful, and even might be trusted to entirely in some cases. Dr. Wood also condemns all depleting treatment, and even purgatives during the first ten days, as not only not required, but positively dangerous, as tending to interfere with the development of the eruption. In the latter stages, as well as in the dropsy, however, he thinks purgatives are often beneficial.

The general plan of this treatment appears to be so far rational that its object is to hurry forward the disease by applying damp heat to the skin, and by thus assisting nature to make her operations more perfect than they might otherwise be. In other words, by rendering the febrile eruption more complete, diminish the risk of its leaving behind it a tendency to subsequent disease. Whether this plan as a whole will, in practice, prove more extensively beneficial than any other, can only be determined by an extensive trial and careful comparison of the results.—*Monthly Journal of Med. Science.*

